# Characteristics and service use of NSW Quitline callers with and without mental health conditions

**DOI:** 10.3389/fpsyt.2022.868084

**Published:** 2022-12-05

**Authors:** Simone Lodge, Kate Bartlem, Lauren Gibson, Caitlin Fehily, Tegan Bradley, Emma McKeon, Kate Reakes, Sandra Rickards, Phillipa Hastings, Jenny Bowman

**Affiliations:** ^1^School of Psychological Sciences, The University of Newcastle, Callaghan, NSW, Australia; ^2^Hunter New England Population Health, Wallsend, NSW, Australia; ^3^Screening and Prevention, Cancer Institute, St Leonards, NSW, Australia; ^4^Hunter Medical Research Institute, New Lambton Heights, NSW, Australia

**Keywords:** smoking, cessation, mental health, Quitline, chronic disease

## Abstract

**Introduction:**

Smoking rates remain higher for people with a mental health condition compared to the general population and contribute to greater chronic disease burden and premature mortality. Quitline services offer telephone-based smoking cessation support to the public and have been shown to be effective. There is limited research exploring the characteristics of smokers with a mental health condition who use the Quitline or the impacts of using the service on their smoking behaviors.

**Methods:**

This observational study aimed to compare demographic and smoking related characteristics, service use and quit attempts of callers to the New South Wales Quitline (2016–2018) with and without a mental health condition (*N* = 4,219).

**Results:**

At baseline, 40% of callers reported a current mental health condition. Desire to quit smoking was similar for both groups, however participants with a mental health condition had higher nicotine dependency and had made more quit attempts prior to engaging with the service. During program enrolment, quit attempts and 24 hours smoke free periods were similar, however participants with a mental health condition engaged in a greater number of calls and over a longer period with Quitline compared to those without.

**Discussion:**

The findings suggest Quitline efficacy for people with a mental health condition in making a quit attempt for at least 24 h. Increasing the use of Quitline services and understanding service use for this critical group of smokers will increase the likelihood that their quit attempts are transformed into sustained periods of smoking abstinence. Future research should explore whether tailoring of Quitline service provision for people with mental health conditions may increase the likelihood of quit success.

## Introduction

Tobacco smoking accounts for more than 8 million deaths globally every year, making it one of the leading causes of preventable death (1), largely due to chronic diseases such as cancer, cardiovascular disease and lung disease (2). The global prevalence of tobacco smoking has declined for the general population from 26.9 in 2000 to 20.2% in 2015 (3). However, for people with a mental health condition smoking prevalence has remained relatively unchanged with higher prevalence associated with more severe mental health conditions e.g., schizophrenia (64%) and bipolar disorder (44%) (4, 5). In Australia, people with a mental health condition live an estimated 12.0 years less for women and 15.9 years less for men compared to the general population (6). This is largely due to chronic diseases of which smoking is a major factor (6). Addressing this gap in life expectancy is recognized as a priority with national, state and local policies implementing strategies to support smoking cessation for this population (7).

Evidence suggests that smokers with a mental health condition are equally likely to want to quit (8) and to attempt to do so (9). Certain characteristics that typify smokers with a mental health condition however (e.g., low socio-economic status, living in smoky environments and high nicotine dependency) (10), may nevertheless make it harder for those who access support for quitting to change their smoking behavior. As a result, it is important to understand how to maximize the effectiveness of cessation supports for this group to increase the likelihood of continued abstinence.

The World Health Organization recommends national toll-free telephone Quitlines as a population level strategy for countries to support smoking cessation (11). Quitline services have a broad reach and have been identified as a cost-effective means of delivering smoking cessation support (12, 13). In Australia, each state government is responsible for Quitline service delivery. Inbound calls to the service incur call costs, with outbound calls delivered at no cost to the participant. The New South Wales (NSW) Quitline service has been in operation since 2002 and is facilitated by the Cancer Institute NSW. The primary aim of the NSW Quitline is to provide smoking cessation support and information to smokers, ex-smokers and the general population. Few studies have explored the characteristics of smokers with a mental health condition who use the Quitline or the benefit they may gain from doing so (14).

To address this gap in the research literature, the study aimed to compare participants with and without a mental health condition with respect to their:

1) demographic and smoking-related characteristics upon enrolment with the NSW Quitline callback service; and
2) service use and changes in smoking behavior (quit attempts and 24 h smoke free periods) during enrolment.

## Materials and methods

### Design and setting

The study involved secondary analysis of data routinely collected as part of service delivery by the NSW Quitline between February 2016 and February 2018. NSW Quitline is staffed by professionals who provide advice, information and support to people seeking help to quit smoking. They can provide one-off calls, or offer an outbound call service, offering up to six “callbacks”, both pre-quit (preparing and setting a quit date), plus calls at 3, 7, 14, and 28 days (monitoring and supporting a quit attempt). Follow up calls are offered at 3, 6, and 12 months (ongoing cessation and relapse support). The number of calls people enrolled in the callback service may receive is determined by the individual. Counselors may discuss how the client can access additional support such as nicotine replacement therapy, or other support services. In 2017–18, the NSW Quitline actively recruited mental health professionals to enhance the services available to people with mental health conditions.

### Participants

For Aim one, eligible participants were current smokers who enrolled in the Quitline telephone callback service during the study period, aged over 18 years and who had provided a response to the mental health condition screening question indicating the presence/absence of a mental health condition. Of the 16,211 callers who contacted the NSW Quitline service during the study period, 4,219 participants met the inclusion criteria. For Aim two, the same criteria applied, however, callers who were still actively enrolled in the program were excluded from the analysis as they had not yet completed the program, and therefore were not appropriate for an examination of change in smoking behavior. During the 2-year study period, 2,253 of the 4,219 enrolled participants had finalized their enrolment (e.g., were no longer actively engaged as they had graduated, withdrawn, suspended or been referred on) in the callback program.

### Data collection procedures and measures

At enrolment, participants were asked “Do you currently have a mental health condition that you have been seeing a doctor about?” (Yes/No). Of those who responded “Yes”, data was collected about specific diagnoses where participants could report more than one mental health diagnosis. Participants were also asked a series of questions relating to demographic and smoking-related related characteristics (see Table 1). At each follow-up call, participants were asked “Did you try to quit?” (No/Yes–failed/Yes–succeeded; defined as a period of 24 h without a cigarette). The number of contacts were documented on the participants' record.

**Table 1:** Comparisons between demographic and smoking-related characteristics of participants enrolled in the NSW Quitline callback service.

	**All** **(*****N*** = **4,219)**		**Mental health condition** **(*****N*** = **1,693)**		**No mental health condition** **(*****N*** = **2,526)**		***p***
	***n***	**%**	***n***	**%**	***n***	***%***	
**Demographic characteristics**							
Gender							<0.001
Male	2,214	52.5	733	43.3	1,481	58.6	
Female	2,004	47.5	959	56.6	1,045	41.1	
Trans/Other^a^	1	0.0	1	0.1			
Age							
18–34	1,171	27.8	499	26.5	722	28.6	<0.001
35–54	1,804	42.8	796	47.0	1,008	39.9	
55+	1,244	29.5	448	26.5	796	31.5	
Mean age (SD)	45.4	(14.7)	44.7	(13.65)	45.8	(15.3)	0.015
Marital Status	(*N =* 1,405)		(*N* = 534)		(*N* = 871)		<0.001
Married/De-facto	660	47.0	189	35.4	471	54.1	
Previously or never married	745	53.0	345	64.5	400	45.9	
Education	(*N* = 3,857)		(*N* = 1,533)		(*N* = 2,324)		0.018
University	634	16.4	221	14.4	413	17.8	
Vocational^b^	760	19.7	320	20.9	440	18.9	
High school or less	2,362	61.2	945	61.6	1,417	61.0	
Employment	(*N* = 4,126)		(*N* = 1,642)		(*N* = 2,484)		<0.001
Employed	2,007	48.6	517	31.5	1,490	60.0	
Unemployed	1,134	27.5	692	42.1	442	17.8	
Retired/home duties/student	734	17.8	286	17.4	448	18.0	
Living arrangements	(*N* = 3,110)		(*N* = 1,318)		(*N* = 1,992)		<0.001
Alone	876	26.5	459	34.8	417	20.9	
Not alone	2,434	73.5	859	65.2	1,575	79.1	
Living environment	(*N* = 2,432)		(*N* = 857)		(*N* = 1,575)		0.083
With smokers	1,038	42.7	386	45.0	652	41.4	
With non-smokers	1,394	57.3	471	55.0	923	58.6	
**Smoking related characteristics**							
Heaviness of Smoking Index (HSI)^c^	(*N* = 3,720)		(*N* = 1,424)		(*N* = 2,296)		<0.001
Low	528	14.2	158	11.1	370	16.1	
Moderate	1,512	40.6	532	37.4	980	42.7	
High	1,680	45.2	734	51.5	946	41.2	
Desire to quit (1–10)^d^	(*N* = 3,652)		(*N* = 1,449)		(*N* = 2,203)		0.024
1–3 (Low)	46	1.3	23	1.6	23	11.9	
4–7 (Medium)	432	11.8	193	13.3	239	10.8	
8–10 (High)	3,174	86.9	1,233	85.1	1,941	88.1	
Previous quit attempts	(*N* = 3,703)		(*N* = 1,497)		(*N* = 2,206)		<0.001
0–2	1,803	48.7	696	46.5	1,107	50.2	
3–4	909	24.5	340	22.7	569	25.8	
5 or more	991	26.8	461	30.8	530	24.0	

Responses of “Declined to answer”, “Null” and missing data are excluded from analyses.

a Trans/Other category excluded from chi-square analysis.

b Includes advanced diploma, associate diploma, certificate II and trade certificate.

c HSI was calculated from the sum of the two responses to CPD and TTFC which were coded as follows: 0 (0–10 CPD; TTFC ≥ 61 min), 1 (11–20 CPD; TTFC, 31–60 min), 2 (21–30 CPD; TTFC, 6–30 min) or 3 (31 CPD; TTFC, 5 min).

A scale from 0 to 6 was used to categorize low (0–1), medium (2–4), and high (5–6) dependency.

d Self-reported on a scale where 1 = not at all, 10 = a lot.

### Statistical analysis

Nicotine dependence (low 0–1, medium 2–4, high 5–6) was calculated using the Heaviness of Smoking Index (HSI) (15), see footnote Table 1. Two smoking cessation variables were calculated based on participant responses to “Did you try to quit?” (No/Yes-failed/Yes-succeeded): (1) Any quit attempt (No vs. Yes–failed, Yes-succeeded) and (2) 24 h smoke free period (Yes–succeeded vs. Yes–failed). Due to this question being asked at each follow-up call, a participant was classified as having made any quit attempt, or having a 24 h smoke free period, if they reported so at any point during the callback service.

Chi-square tests were conducted to compare demographic and smoking related characteristics, and a *t*-test to compare mean number of calls, between participants with and without a mental health condition. Logistic regression analyses were undertaken to examine whether self-reported mental health condition (yes/no) was associated with either making a quit attempt or sustaining a 24 h smoke free period during enrolment with the service, when adjusting for variables that may impact smoking outcomes (education, employment, heaviness of smoking index) (16). Due to the large sample size and multiple tests being undertaken, the threshold for statistical significance was *p* < 0.01. Analyses included all available data.

## Results

### Mental health conditions

Of the 4,219 enrolled participants, 40.1% self-reported the presence of a mental health condition. Depression was the most frequently reported diagnosis (47.2%), followed by anxiety disorder (33.1%), schizophrenia/schizoaffective disorder (17.2%) and bipolar disorder (13.5%).

### Demographic and smoking-related characteristics of enrolled participants

Participants with a mental health condition were significantly more likely to be female (56.6 vs. 41.1%), unemployed (42.1 vs. 17.8%), living alone (34.8 vs. 20.9%), and less likely to be married/partnered (35.4 vs. 54.1%) or living in a smoke-free environment (55.0 vs. 58.6%) compared to participants without a mental health condition.

There were no significant differences in desire to quit smoking at enrolment for participants with and without a mental health condition. Participants with a mental health condition were significantly more likely to report a high nicotine dependency (51.5 vs. 41.2%) and to have made more than five quit attempts prior to enrolment (30.8 vs. 24.0%) compared to those without a mental health condition (see Table 1).

### Service use and smoking outcomes of finalized enrolments

#### Service use

During enrolment, participants with a mental health condition engaged in a significantly greater number of calls with Quitline callback services (M = 5.7, SD = 8.3, range = 178) compared to participants without a mental health condition (M = 4.4, SD = 3.3, range = 32), *p* < 0.001. Similarly, length of treatment (e.g., number of days from first to last call) was greater for participants with a mental health condition (M = 81, SD = 135, range = 745) compared to those without (M = 64, SD = 103, range = 621).

#### Quit attempts and 24 h smoke free periods

During Quitline enrolment, there were no significant associations between participants mental health condition status (yes/no) and the likelihood of having made at least one quit attempt or having achieved at least one smoke free 24 h period (Table 2). Of those with available data, 75% of participants with a mental health condition made a quit attempt, compared to 71.9% of those without a mental health condition. Likewise, 57.9% of participants with a mental health condition had a 24 h smoke free period, compared to 66.7% of those without.

**Table 2:** Comparisons between mental health conditions, quit attempts and 24 h smoke free periods for finalized participants in the NSW Quitline callback service.

	**All** **(*****N*** = **2,253)**		**Mental health condition** **(*****N*** = **957)**		**No mental health condition** **(*****N*** = **1,295)**		**OR [95% CI]**	***p***
	***n***	**%**	***n***	**%**	***n***	***%***		
**Smoking outcomes**								
Quit attempt^a^	(*N* = 550)		(*N* = 212)		(*N* = 338)			0.349
Yes	402	73.1	159	75.0	243	71.9	0.81 [0.52, 1.26]	
No	148	26.9	53	25.0	95	28.1	1.00	
24 h smoke free period^b^	(*N =* 402)		(*N* = 159)		(*N* = 243)			0.136
Yes	254	63.2	92	57.9	162	66.7	1.4 [0.89, 2.30]	
No	148	36.8	67	42.1	81	33.3	1.00	

a Responses to the question “Did you try to quit” were used to calculate quit attempts and included participant responses “No”, “Yes-Failed” or “Yes-Succeeded”, where succeeded is defined as a smoke free period of at least 24 h.

b Responses to the question “Did you try to quit” were used to calculate 24 h smoke free quit attempts and included participant responses “Yes-Failed” or “Yes-Succeeded”.

Models adjusted for education, employment, and heaviness of smoking index.

Reference = no mental health condition.

## Discussion

To the authors' knowledge, this is the first study to explore use of the NSW Quitline, and changes to smoking behavior, for people with a mental health condition. Among all enrolments, participants with a mental health condition were just as likely to want to quit smoking as participants without a mental health condition. Among finalized enrolments, those with a mental health condition were just as likely to report a quit attempt, and to have at least one such attempt result in a smoke free period of at least 24 h as those without mental health conditions.

Of the 4,219 participants in the study, more than a third (40.1%) self-reported the presence of a mental health condition; most commonly depression, followed by anxiety. Consistent with findings from previous Quitline studies in Australia (10), participants with a mental health condition were more likely to be single, unemployed or live with other smokers when compared to participants without a mental health condition. Consistent with previous international research (17), participants with a mental health condition tended to smoke more cigarettes daily and were more likely to have a high nicotine dependence, factors which mitigate against the likelihood of quitting success.

Participants with a mental health condition had made a greater number of quit attempts prior to enrolment compared to participants without a mental health condition. The present research stands in contrast to the documented beliefs among some health clinicians that those with a mental health condition are not interested in quitting (18). It is important to address health clinicians' beliefs about this population (as they often make referrals to Quitline) to ensure they know there is a desire to quit and that, when offered support to do so, people with a mental health condition are likely to make at least short-term changes to smoking behavior.

The similar number of quit attempts and 24 h periods of abstinence during enrolment suggests that both participants with and without mental health conditions received adequate levels of support from the Quitline callback service to make at least short-term changes in their smoking behavior. However, participants with a mental health condition engaged in significantly more telephone calls with the service over a longer period when compared to participants without a mental health condition. This finding is consistent with previous research (19) and suggests that people with a mental health condition may require additional support when making a quit attempt due to additional barriers such as higher nicotine dependence and limited social support. This could have implications for Quitline services to work toward tailoring, through strategies such as routine provision of nicotine replacement treatment starter packs to help address cessation challenges such as more severe withdrawal, financial hardship and social isolation. Some studies have attempted to address these barriers through trialing different models of smoking cessation care that include monitoring the effects of nicotine withdrawal during a quit attempt (20) and utilizing peer workers to provide encouragement (21). Further research is required to examine the optimal level and type of support Quitline services might provide to people with mental health conditions to increase the likelihood of quit success and effectiveness in supporting long term cessation.

Limitations of the study include the capacity to look at only short term changes in smoking behavior, such as a 24 h period of abstinence as opposed to a measure such as 7-day point prevalence; although, 24 h abstinence is a measure commonly used in tobacco research (22). A lack of participant data at follow-up meant it was not possible to report on long-term cessation attempts. Challenges of real-world data collection and using service data not primarily designed to address research questions, contributed to such instances of missing data and difficulty in discerning the underlying reasons—such as participant drop out from the program, lack of rigor in data collection or other factors. The individualized nature of Quitline service delivery and resultant variation in data collection points led to some inconsistency with respect to “when” data pertaining to changes in smoking behavior was collected. It should also be acknowledged that during the study period the Quitline service began to employ some staff with mental health qualifications. Exploring to what extent participants with a MHC received their calls from a mental health professional, and possible associations of having a mental health advisor with service use and cessation related outcomes, would be valuable to address in future research.

Smokers with a mental health condition engaging with the NSW Quitline experienced high nicotine dependence, were more likely to be living in smoky environments and less likely to have partner support. Despite this, such smokers who commence with the Quitline service are equally likely to want to quit and report having made more previous attempts to do so than people without a mental health condition. Furthermore, people with a mental health condition are equally likely to make a quit attempt and cease smoking for at least 24 h whilst enrolled when compared to smokers without a mental health condition. Further research is required to understand long-term smoking outcomes of people with mental health conditions, and whether tailoring of Quitline service provision to consider the needs and characteristics of this group of smokers will increase the likelihood that quit attempts are translated into smoking cessation.

## Data availability statement

The data analyzed in this study is subject to the following licenses/restrictions: The data is stored electronically in a confidential file on a password-protected computer at the School of Psychological Sciences at the University of Newcastle. Data published contains aggregate results only. Requests to access these datasets should be directed to Simone Lodge, simone.lodge@newcastle.edu.au.

## Ethics statement

The studies involving human participants were reviewed and approved by NSW Population and Health Services Research Ethics Committee 2018HRE0702. Written informed consent for participation was not required for this study in accordance with the national legislation and the institutional requirements.

## Author contributions

JB and KB: conceptualization, methodology, validation, and supervision. SL: formal analysis, investigation, writing—original draft. LG: conceptualization, methodology, formal analysis, and writing—review and editing. CF and TB: formal analysis and writing—review and editing. EM: investigation and formal analysis. KR, SR, and PH: writing—review & editing. All authors contributed to the article and approved the submitted version.

## Funding

KB is supported by a National Health and Medical Research Council Early Career Fellowship (#1142272).

## Conflict of interest

The authors declare that the research was conducted in the absence of any commercial or financial relationships that could be construed as a potential conflict of interest.

## Publisher's note

All claims expressed in this article are solely those of the authors and do not necessarily represent those of their affiliated organizations, or those of the publisher, the editors and the reviewers. Any product that may be evaluated in this article, or claim that may be made by its manufacturer, is not guaranteed or endorsed by the publisher.
